# Wave Dispersion Behaviors of Multi-Scale CNT/Glass Fiber/Polymer Nanocomposite Laminated Plates

**DOI:** 10.3390/polym14245448

**Published:** 2022-12-13

**Authors:** Farzad Ebrahimi, Alireza Enferadi, Ali Dabbagh

**Affiliations:** 1 Department of Mechanical Engineering, Faculty of Engineering, Imam Khomeini International University, Qazvin 34148-96818, Iran; 2 School of Mechanical Engineering, College of Engineering, University of Tehran, Tehran 11155-4563, Iran

**Keywords:** wave propagation, multi-scale hybrid nanocomposite, laminated composite, refined plate theory

## Abstract

In this paper, wave propagation in multi-scale hybrid glass fiber (GF)/carbon nanotube (CNT)/polymer nanocomposite plates is studied for the first time by means of refined higher-order plate theory. The hybrid nanocomposite consists of CNTs and glass fibers (GF) as reinforcing components distributed within a polymeric matrix. A hierarchical micromechanical approach is used to predict the effective mechanical properties of the hybrid nanocomposite, including the three-dimensional (3D) Mori-Tanaka method and the rule of mixture. Moreover, a refined-type higher-order shear deformation theory (HSDT) is implemented to take into account the influence of the shear deformation on the motion equations of the system. Then, the governing equations are achieved on the basis of the energy-based Hamilton’s principle. Finally, the derived equations will be solved analytically for the purpose of extracting the natural frequency of the continuous system. A set of numerical examples are provided to cover the effects of various parameters on the wave dispersion characteristics of the plate. It can be declared that the hybrid nanocomposite system can achieve higher wave frequencies compared with other types of composite structures. Additionally, it is found that the selection of the lay-ups and length-to-diameter ratio plays a significant role in the determination of the sandwich plate’s acoustic response.

## 1. Introduction

Nanocomposites are an extraordinary class of advanced materials that have emerged as suitable alternatives to overcome the limitations of traditional ones due to their variety of merits. These innovative materials have many magnificent properties such as low cost-to-performance ratio, high specific stiffness and strength, incredible fire retardancy, enhanced corrosion resistance and improved fatigue life. Therefore, they can meet the design standards in many aspects of engineering fields, e.g., mechanics, aerospace, naval and marine [1]. Another feature that makes nanocomposite materials attractive is the fact that nanosize reinforcements have high surface-to-volume ratios that contribute to a better energy transfer from the matrix to the reinforcement phase, and vice versa [2]. Supplementary to these, their rection upon exposure to water is another particular aspect of nanomaterials of this type [3].

During the last decade, a new type of nanocomposite was found. This type of nanocomposite can exhibit a combined behavior of both macro- and nano-composites. The aforementioned class of nanocomposite, also known as multi-scale hybrid (MSH) nanocomposite, can demonstrate far better material properties than traditional nanocomposites do [4]. Thus, many researchers have focused on investigating the mechanical behaviors of structures consisted of such advanced materials. Rafiee et al. [5] surveyed the free vibration problem of the piezoelectric nanotubes/fiber/polymer multi-scale laminated composite plates considering the influence of geometrical nonlinearity in the framework of the Timoshenko beam hypothesis. Then, He et al. [6] developed a fractional-order time derivative damping model to study the damped dynamic characteristics of viscoelastic MSH nanocomposite beams. Additionally, Rafiee et al. [7] utilized the Halpin-Tsai homogenization technique to explain the static and dynamic responses of rotary MSH nanocomposite thin-walled airfoils. In another study, Ahmadi et al. [8] surveyed the bending, static stability, and vibration problems of MSH nanocomposite plates of various shapes using the finite element method (FEM). Afterward, Hassanzadeh-Aghdam et al. [9] found that the thermal conductivity of MSH nanocomposites can be much better than those of conventional composites. The influence of the CNTs waviness was discussed in the aforementioned report. Moreover, the effect of agglomerates of silicone carbide on the tensile behaviors of MSH nanocomposite materials was investigated. Moreover, it was reported that by adding SiC nanoparticles, the elastic modulus of the SiC whisker-reinforced composites can be enhanced [10]. Ebrahimi and Habibi [11] utilized FEM to determine the response of MSH nanocomposite rectangular plates rested on elastic foundations to low-velocity impact input in which both temperature and moisture gradients were taken into consideration. In addition, Rafiee et al. [12] examined both static and dynamic characteristics of MSH nanocomposite beams made of graphene platelets/fiber/polymer within the framework of the Euler-Bernoulli beam theory. Moreover, Ebrahimi and Dabbagh [13] investigated the natural frequency response of MSH nanocomposite beams in a thermal environment on the basis of a higher-order shear deformation theory (HSDT). The thermal effect on the MSH annular plates was solved by Safarpour et al. [14] using the generalized differential quadrature method (GDQM). In addition, the vibration characteristics and nonlinear frequency of the MSH disks and plates embedded in a viscoelastic media were investigated [15,16]. In all the studies mentioned so far, carbon fiber (CF) has been used mainly as the reinforcing component in macroscale.

The issue of studying the mechanical behavior of MSH nanocomposites including glass fibers (GFs) has recently been studied. In addition, all of the available works in this area are related to the buckling and vibration responses of such nanocomposite structures [17,18,19,20,21]. Despite the fact that no research has been conducted regarding the wave dispersion characteristics of these nanocomposites, the applications and high importance of this issue in different aspects of engineering cannot be ignored, including the wide usage of this issue in the field of non-destructive tests [22]. Therefore, in order to compensate for this deficiency, the wave propagation curves of MSH (CNT/GF/polymer) nanocomposite plates is carried out as a novel research in the context of the analytical method introduced in [22]. This work is the first in this field which can provide practical insights for the aid of designing and manufacturing many acoustical instruments for sensing defects in solids.

The next section is devoted to mathematical modeling of the problem. First, a general explanation of the problem is given. Next, the equivalent material properties will be enriched using the micromechanical homogenization scheme. Then, the governing equations are obtained based on the dynamic form of the virtual work’s principle. Afterward, the corresponding eigenvalue equations will be solved by the means of the well-known exponential analytical wave method. Finally, numerical examples are included to illustrate physical trends. This multi-step modeling allows any individual to realize the derivation procedure in terms of some separated stages. In addition, the presented formulations are valid for the free vibration problem of the same topic which makes the modeling a general one. Additionally, the utilized analytical solution has a vast range of benefits, the most important of which is its capability to reflect the wave response of the continuous system with the least possible computational cost.

## 2. Theory and Formulation

### 2.1. Problem Definition

**Figure 1:**
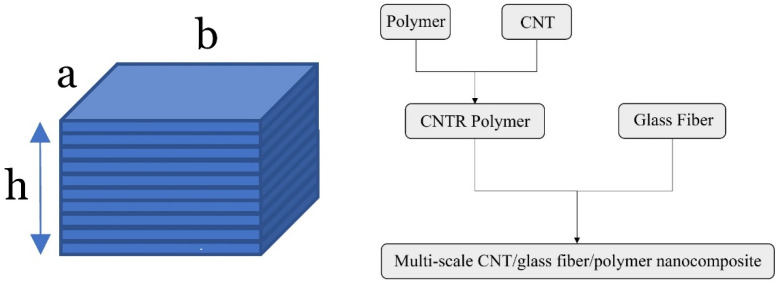
Schematic view of the analyzed multi-layered plate (**left** figure) and the order by which the ingredients must be combined to manufacture a single ply of hybrid nanocomposite material (**right** figure).

In present study the problem of elastic wave dispersion within plate-type elements of length *a*, width *b*, and thickness *h* (shown in Figure 1) is solved. It is noteworthy that the structure is reinforced with a mixture of multi-scale reinforcing gadgets, CNTs and GF as nanoscale and macroscale reinforcements, respectively. For a better understanding of how these elements are mixed in each ply of the laminate, the readers are referred to Figure 1. In this study, the case of analyzing thick plates (i.e., corresponding with *a*/*h* = 10) will be considered due to the fact that refined-type HSDT is utilized here which is able to predict reliable answers for plates with low length-to-thickness ratio. Moreover, in all the numerical examples, the plate’s thickness is considered to be h=2 mm.

### 2.2. Homogenization Procedure

In this section, the effective mechanical properties of the GF/CNT/polymer nanocomposite will be attained based on a two-step micromechanical scheme. Since the assumption that the nanotubes in the polymer nanocomposite are aligned with each other is far from reality, the 3D Mori-Tanaka method with the goal of presenting near-real numerical results is used to obtain equivalent material properties of the CNTR polymer nanocomposite. Then, the concept of the rule of the mixture will be utilized in order to calculate the material properties of the GF/CNT/polymer hybrid nanomaterial. In view of the broad explanation of the second step in [23,24,25,26,27], the attention of this section is focused on examining the first step. Based on [28], elastic, shear, and bulk modules of CNTR nanocomposites can be expressed in the following form:

(1) $$\frac{E_{11}}{E_{m}}=\frac{1}{1+\frac{V_{CNT}\left(Z_{1}+2\mu_{m}Z_{2}\right)}{\tilde{Z}}}$$

(2) $$\frac{E_{22}}{E_{m}}=\frac{1}{1+\frac{V_{CNT}\left(\left[1-\mu_{m}\right]Z_{4}-2\mu_{m}Z_{3}+\left[1+\mu_{m}\right]Z_{5}\tilde{Z}\right)}{2\tilde{Z}}}$$

(3) $$G_{12}=G_{m}+\frac{G_{m}V_{CNT}}{\frac{G_{m}}{\Delta G}+2\left(1-V_{CNT}\right)S_{1212}}$$

(4) $$G_{23}=G_{m}+\frac{G_{m}V_{CNT}}{\frac{G_{m}}{\Delta G}+2\left(1-V_{CNT}\right)S_{2323}}$$

(5) $$K_{23}=\frac{\left(G_{m}+\Gamma_{m}\right)\left(1+\mu_{m}\right)\left(1-2\mu_{m}\right)}{1-\mu_{m}\left(1+2\mu_{12}\right)+\frac{V_{CNT}}{\tilde{Z}}\left(2\left[\mu_{12}-\mu_{m}\right]Z_{3}+\left[1-\mu_{m}\left(1+2\mu_{12}\right)\right]Z_{4}\right)}$$

in which $G_{m}$ is the matrix’s shear modulus. Additionally, $E_{m}$ and $\mu_{m}$ stand for Young’s modulus and Poisson’s ratio of the matrix, respectively. Moreover, ΔG is the difference between the shear modules of matrix and CNT and will be considered as $\Delta G=G_{CNT}-G_{m}$. The term $\mu_{12}$ reflects in-plane Poisson’s ratio of the three-phase nanocomposite. In the above equations, the volume fraction of the CNTs can be presented in the following form [25]:

(6) $$V_{CNT}=\frac{W_{CNT}\rho_{m}}{\left(1-W_{CNT}\right)\rho_{CNT}+W_{CNT}\rho_{m}}$$

where $W_{CNT}$ represents the weight fraction of the CNTs. In addition, $\rho_{m}$ and $\rho_{CNT}$ are mass densities of matrix and CNT, respectively. Next, the parameters $Z_{i}$ in Equations (1)–(5) can be described in the following form [20,28]:

(7) $$\begin{array}{l} Z_{1}=D_{1}\left(L_{4}+L_{5}\right)-2L_{2},\\ Z_{2}=\left(1+D_{1}\right)L_{2}-\left(L_{4}+L_{5}\right),\\ Z_{3}=L_{1}-D_{1}L_{3},\\ Z_{4}=\left(1+D_{1}\right)L_{1}-2L_{3},\\ Z_{5}=\frac{1-D_{1}}{L_{4}-L_{5}},\\ \tilde{Z}=2L_{2}L_{3}-L_{1}\left(L_{4}+L_{5}\right) \end{array}$$

Where

(8) $$\begin{array}{l} L_{1}=V_{CNT}D_{1}+D_{2}+\left(1-V_{CNT}\right)\left(D_{1}S_{1111}+2S_{2211}\right),\\ L_{2}=V_{CNT}+D_{3}+\left(1-V_{CNT}\right)\left(D_{1}S_{1122}+S_{2222}+S_{2233}\right),\\ L_{3}=V_{CNT}+D_{3}+\left(1-V_{CNT}\right)\left(S_{1111}+\left[1+D_{1}\right]S_{2211}\right),\\ L_{4}=V_{CNT}D_{1}+D_{2}+\left(1-V_{CNT}\right)\left(S_{1122}+D_{1}S_{2222}+S_{2233}\right),\\ L_{5}=V_{CNT}+D_{3}+\left(1-V_{CNT}\right)\left(S_{1122}+S_{2222}+D_{1}S_{2233}\right) \end{array}$$

In the aforementioned equations, $S_{ijkl}$ arrays denote the components of the 4th-order Eshelby tensor which can be found in Appendix A. In the above definitions $D_{i}$’s can be defined as following [20]:

(9) $$D_{1}=1+\frac{2\Delta G}{\Gamma_{CNT}-\Gamma_{m}},\;\;\;\;\;D_{2}=\frac{\Gamma_{m}+2G_{m}}{\Gamma_{CNT}-\Gamma_{m}},\;\;\;\;\;D_{3}=\frac{\Gamma_{m}}{\Gamma_{CNT}-\Gamma_{m}}$$

in which Lame’s constants of matrix ($\Gamma_{m}$) and CNT ($\Gamma_{CNT}$) can be described as [20,29]:

(10) $$\Gamma_{m}=\frac{\mu_{m}E_{m}}{\left(1-2\mu_{m}\right)\left(1+\mu_{m}\right)},\;\;\;\;\;\;\;\;\Gamma_{CNT}=\frac{\mu_{CNT}E_{CNT}}{\left(1-2\mu_{CNT}\right)\left(1+\mu_{CNT}\right)}$$

where $E_{CNT}$ and $\mu_{CNT}$ stand for Young’s modulus and Poisson’s ratio of the CNT, respectively. Eventually, the effective bulk ($\hat{K}$) and shear ($\hat{G}$) modules of CNTR nanocomposite can be calculated using the below definitions [20]:

(11) $$\hat{K}=\frac{E_{11}+4{\left(1+\mu_{12}\right)}^{2}K_{23}}{9}$$

(12) $$\hat{G}=\frac{E_{11}+{\left(1-2\mu_{12}\right)}^{2}K_{23}+6\left(G_{12}+G_{23}\right)}{15}$$

Using the above-mentioned relationships and considering the relation between bulk modulus, shear modulus, Young’s modulus and Poisson’s ratio in a linearly elastic isotropic solid [29], Young’s modulus and the Poisson’s ratio of the CNTR polymer nanocomposite can be determined using the definitions below [20]:

(13) $$\hat{E}=\frac{9\hat{K}\hat{G}}{3\hat{K}+\hat{G}}\;\;\;,\;\;\hat{\mu }=\frac{\hat{E}}{2\hat{G}}-1$$

Now, we easily apply the procedure explained by the authors in their previous works to obtain the mechanical properties for the MSH GF/CNT/polymer nanocomposite [27]. It is worth mentioning that the mass density of the MSH nanocomposite can be achieved via the extended rule of the mixture for a hybrid three-phase material.

### 2.3. Refined Higher-Order Plate Theory

This part deals with the introduction of the kinematic theory, which is used to obtain the governing equations of the problem. To date, several types of theories for beam, plate, and shell geometries have been developed by researchers, and a review of some of the well-known theories is provided in [29]. Due to the fact that the classical theories disregard shear strain and stress on the deflection of thick-type structures, the responses obtained from these theories have errors and they cannot be used for the cases of which the mentioned structures are going to be studied. In this research, a refined-type HSDT will be used in order to make it possible to investigate the dynamic behaviors of the thick plate accurately. According to this theory, the displacement field of a plate can be written as [27]:

(14) $$\begin{array}{l} u_{x}(x,y,z,t)=u(x,y,t)-z\frac{\partial w_{b}(x,y,t)}{\partial x}-f(z)\frac{\partial w_{s}(x,y,t)}{\partial x},\\ u_{y}(x,y,z,t)=v(x,y,t)-z\frac{\partial w_{b}(x,y,t)}{\partial x}-f(z)\frac{\partial w_{s}(x,y,t)}{\partial x},\\ u_{z}(x,y,z,t)=w_{b}(x,y,t)+w_{s}(x,y,t) \end{array}$$

where u and v denote the longitudinal and transverse displacements of the mid-surface, respectively; also, $w_{b}$ and $w_{s}$ stand for the bending and shear deflections through z-axis, respectively. Moreover, the corresponding shape function of the employed theory is as same as that introduced in [30]. In order to reach the nonzero components of the Cauchy strain tensor, the concept of the infinitesimal strains of a continuous system in Cartesian coordinate system and displacement field given in Equation (14) are used. Now, the nonzero strains of the plate can be calculated as follows [27]:

(15) $$\left\{\begin{array}{c} \varepsilon_{xx}\\ \varepsilon_{yy}\\ \gamma_{xy} \end{array}\right\}=\left\{\begin{array}{c} \varepsilon_{xx}^{0}\\ \varepsilon_{yy}^{0}\\ \gamma_{xy}^{0} \end{array}\right\}+z\left\{\begin{array}{c} \kappa_{xx}^{b}\\ \kappa_{yy}^{b}\\ \kappa_{xy}^{b} \end{array}\right\}+f(z)\left\{\begin{array}{c} \kappa_{xx}^{s}\\ \kappa_{yy}^{s}\\ \kappa_{xy}^{s} \end{array}\right\},\;\;\;\;\;\;\;\;\left\{\begin{array}{c} \gamma_{xz}\\ \gamma_{yz} \end{array}\right\}=g(z)\left\{\begin{array}{c} \gamma_{xz}^{0}\\ \gamma_{yz}^{0} \end{array}\right\}$$

Where

(16) $$\left\{\begin{array}{c} \varepsilon_{xx}^{0}\\ \varepsilon_{yy}^{0}\\ \gamma_{xy}^{0} \end{array}\right\}=\left\{\begin{array}{c} \frac{\partial u}{\partial x}\\ \frac{\partial v}{\partial y}\\ \frac{\partial u}{\partial x}+\frac{\partial v}{\partial y} \end{array}\right\},\;\;\;\;\left\{\begin{array}{c} \kappa_{xx}^{b}\\ \kappa_{yy}^{b}\\ \kappa_{xy}^{b} \end{array}\right\}=\left\{\begin{array}{c} -\frac{\partial^{2}w_{b}}{\partial x^{2}}\\ -\frac{\partial^{2}w_{b}}{\partial y^{2}}\\ -2\frac{\partial^{2}w_{b}}{\partial x\partial y} \end{array}\right\},\;\;\;\;\left\{\begin{array}{c} \kappa_{xx}^{s}\\ \kappa_{yy}^{s}\\ \kappa_{xy}^{s} \end{array}\right\}=\left\{\begin{array}{c} -\frac{\partial^{2}w_{s}}{\partial x^{2}}\\ -\frac{\partial^{2}w_{s}}{\partial y^{2}}\\ -2\frac{\partial^{2}w_{s}}{\partial x\partial y} \end{array}\right\},\;\;\;\;\;\left\{\begin{array}{c} \gamma_{xz}^{0}\\ \gamma_{yz}^{0} \end{array}\right\}=\left\{\begin{array}{c} \frac{\partial w_{s}}{\partial x}\\ \frac{\partial w_{s}}{\partial y} \end{array}\right\}$$

### 2.4. Motion Equations

Here the structure’s motion equations, also known as Euler-Lagrange equations, are derived using an energy-based approach. This method (i.e., Hamilton’s principle) states that the total energy change of a continuous system in each time interval must be equal to zero. For that reason, the variation in the system’s Lagrangian will be set to zero. The definition of Lagrangian can be described as below [31]:

(17) L=U−T+V

in which U and T denote strain and kinetic energies, respectively; also, V denotes the work exerted by external loading. Therefore, the principle equation to be considered for achieving the Euler-Lagrange equations of the system is [31]:

(18) $$\delta \int_{t_{1}}^{t_{2}}\left(U-T+V\right)dt=0$$

Next, the variation in strain energy must be determined for a linearly elastic solid. According to the definition, the earlier mentioned variation can be described in the following manner [31]:

(19) $$\delta U=\int_{\forall }\sigma_{ij}\delta \varepsilon_{ij}d\forall$$

where ∀ is the total volume of the continuous structure. Substitution of nonzero strains from Equation (15) in the above equation, the variation in the strain energy of the HSDT-modeled plate can be rewritten as shown below [27]:

(20) $$\begin{array}{cc} \delta U & =\int_{\forall }\left(\sigma_{xx}\delta \varepsilon_{xx}+\sigma_{yy}\delta \varepsilon_{yy}+\sigma_{xy}\delta \gamma_{xy}+\sigma_{xz}\delta \gamma_{xz}+\sigma_{yz}\delta \gamma_{yz}\right)d\forall \\ & =\int_{0}^{b}\int_{0}^{a}\left(\begin{array}{c} N_{xx}\delta \varepsilon_{xx}^{0}+M_{xx}^{b}\delta \kappa_{xx}^{b}+M_{xx}^{s}\delta \kappa_{xx}^{s}+N_{yy}\delta \varepsilon_{yy}^{0}\\ M_{yy}^{b}\delta \kappa_{yy}^{b}+M_{yy}^{s}\delta \kappa_{yy}^{s}+N_{xy}\delta \gamma_{xy}^{0}+M_{xy}^{b}\delta \kappa_{xy}^{b}\\ M_{xy}^{s}\delta \kappa_{xy}^{s}+Q_{xz}\delta \gamma_{xz}^{0}+Q_{yz}\delta \gamma_{yz}^{0} \end{array}\right)dxdy \end{array}$$

in the above equation, the stress-resultants ($N_{ij}$, $M_{ij}^{b}$, $M_{ij}^{s}$, and Q) can be defined as [27]:

(21) $$\begin{array}{cc} \left(N_{ij},M_{ij}^{b},M_{ij}^{s}\right) & =\sum_{k=1}^{N}\int_{h_{k}}^{h_{k+1}}\left(1,z,f\left(z\right)\right)\sigma_{ij}dz,\;\;\;\;\;\left(i,j=x,y\right)\;,\\ Q_{l} & =\sum_{k=1}^{N}\int_{h_{k}}^{h_{k+1}}g\left(z\right)\sigma_{l}dz,\;\;\;\;\left(l=xz,yz\right)\; \end{array}$$

in which g(z)=1−df(z)/dz. Now, it is time to determine first variation in the kinetic energy of the system. Generally, the variation in the kinetic energy of a plate can be defined as below [27]:

(22) $$\delta T=\int_{\forall }\rho \left(\frac{\partial u_{x}}{\partial t}\frac{\partial \delta u_{x}}{\partial t}+\frac{\partial u_{y}}{\partial t}\frac{\partial \delta u_{y}}{\partial t}+\frac{\partial u_{z}}{\partial t}\frac{\partial \delta u_{z}}{\partial t}\right)d\forall$$

Now, the expression of kinetic energy can be extended by substituting Equation (14) in Equation (22):

(23) $$\delta T=\int_{0}^{b}\int_{0}^{a}\left(\begin{array}{c} I_{0}\;\left(\frac{\partial u}{\partial t}\frac{\partial \delta u}{\partial t}+\frac{\partial v}{\partial t}\frac{\partial \delta v}{\partial t}+\frac{\partial w}{\partial t}\frac{\partial \delta w}{\partial t}\right)\\ -I_{1}\left(\frac{\partial u}{\partial t}\frac{\partial^{2}\delta w_{b}}{\partial x\partial t}+\frac{\partial^{2}w_{b}}{\partial x\partial t}\frac{\partial \delta u}{\partial t}+\frac{\partial v}{\partial t}\frac{\partial^{2}\delta w_{b}}{\partial y\partial t}+\frac{\partial^{2}w_{b}}{\partial y\partial t}\frac{\partial \delta v}{\partial t}\right)\\ -J_{1}\left(\frac{\partial u}{\partial t}\frac{\partial^{2}\delta w_{s}}{\partial x\partial t}+\frac{\partial^{2}w_{s}}{\partial x\partial t}\frac{\partial \delta u}{\partial t}+\frac{\partial v}{\partial t}\frac{\partial^{2}\delta w_{s}}{\partial y\partial t}+\frac{\partial^{2}w_{s}}{\partial y\partial t}\frac{\partial \delta v}{\partial t}\right)\\ +I_{2}\left(\frac{\partial^{2}w_{b}}{\partial x\partial t}\frac{\partial^{2}\delta w_{b}}{\partial x\partial t}+\frac{\partial^{2}w_{b}}{\partial y\partial t}\frac{\partial^{2}\delta w_{b}}{\partial y\partial t}\right)\\ +K_{2}\left(\frac{\partial^{2}w_{s}}{\partial x\partial t}\frac{\partial^{2}\delta w_{s}}{\partial x\partial t}+\frac{\partial^{2}w_{s}}{\partial y\partial t}\frac{\partial^{2}\delta w_{s}}{\partial y\partial t}\right)\\ +J_{2}\left(\frac{\partial^{2}w_{b}}{\partial x\partial t}\frac{\partial^{2}\delta w_{s}}{\partial x\partial t}+\frac{\partial^{2}w_{s}}{\partial x\partial t}\frac{\partial^{2}\delta w_{b}}{\partial x\partial t}+\frac{\partial^{2}w_{b}}{\partial y\partial t}\frac{\partial^{2}\delta w_{s}}{\partial y\partial t}+\frac{\partial^{2}w_{s}}{\partial y\partial t}\frac{\partial^{2}\delta w_{b}}{\partial y\partial t}\right) \end{array}\right)dxdy$$

In the above equation, the mass moments of inertia are given in the following form:

(24) $$\left[I_{0},I_{1},I_{2},J_{1},J_{2},K_{2}\right]=\sum_{k=1}^{N}\int_{h_{k}}^{h_{k+1}}\left[1,z,f\left(z\right),z^{2},zf\left(z\right),f^{2}\left(z\right)\right]\rho dz$$

Since there is no external load applied to the structure; the Euler-Lagrange equations can be attained once Equations (23) and (20) substituted in Equation (18). By making the above substitution and choosing the non-trivial response of the achieved equation, the Euler-Lagrange equations can be expressed as below:

(25) $$\frac{\partial Nxx}{\partial x}+\frac{\partial Nxy}{\partial y}=I_{0}\frac{\partial^{2}u}{\partial t^{2}}-I_{1}\frac{\partial^{3}w_{b}}{\partial x\partial t^{2}}-J_{1}\frac{\partial^{3}w_{s}}{\partial x\partial t^{2}},$$

(26) $$\frac{\partial Nxy}{\partial x}+\frac{\partial Nyy}{\partial y}=I_{0}\frac{\partial^{2}v}{\partial t^{2}}-I_{1}\frac{\partial^{3}w_{b}}{\partial y\partial t^{2}}-J_{1}\frac{\partial^{3}w_{s}}{\partial y\partial t^{2}},$$

(27) $$\begin{array}{l} \frac{\partial^{2}M_{xx}^{b}}{\partial x^{2}}+2\frac{\partial^{2}M_{xy}^{b}}{\partial x\partial y}+\frac{\partial^{2}M_{yy}^{b}}{\partial y^{2}}=I_{0}\frac{\partial^{2}\left(w_{b}+w_{s}\right)}{\partial t^{2}}+I_{1}\left(\frac{\partial^{3}u}{\partial x\partial t^{2}}+\frac{\partial^{3}v}{\partial y\partial t^{2}}\right)\\ -I_{2}\left(\frac{\partial^{4}w_{b}}{\partial x^{2}\partial t^{2}}+\frac{\partial^{4}w_{b}}{\partial x^{2}\partial t^{2}}\right)-J_{2}\left(\frac{\partial^{4}w_{s}}{\partial x^{2}\partial t^{2}}+\frac{\partial^{4}w_{s}}{\partial y^{2}\partial t^{2}}\right), \end{array}$$

(28) $$\begin{array}{l} \frac{\partial^{2}M_{xx}^{s}}{\partial x^{2}}+2\frac{\partial^{2}M_{xy}^{s}}{\partial x\partial y}+\frac{\partial^{2}M_{yy}^{s}}{\partial y^{2}}+\frac{\partial Q_{xz}}{\partial x}+\frac{\partial Q_{yz}}{\partial y}=I_{0}\frac{\partial^{2}\left(w_{b}+w_{s}\right)}{\partial t^{2}}+\\ J_{1}\left(\frac{\partial^{3}u}{\partial x\partial t^{2}}+\frac{\partial^{3}v}{\partial y\partial t^{2}}\right)-J_{2}\left(\frac{\partial^{4}w_{b}}{\partial x^{2}\partial t^{2}}+\frac{\partial^{4}w_{b}}{\partial x^{2}\partial t^{2}}\right)-K_{2}\left(\frac{\partial^{4}w_{s}}{\partial x^{2}\partial t^{2}}+\frac{\partial^{4}w_{s}}{\partial y^{2}\partial t^{2}}\right) \end{array}$$

### 2.5. Constitutive Equations

In the previous section, the equations of motion were extracted. Then, it remains to be seen how Equations (25)–(28) can relate to the nanocomposite material under study. For this intention, the constitutive equation of the GF/CNT/polymer material must be entered in the stress resultants. Since the behavior of multiscale hybrid nanocomposites is similar to linear elastic isotropic solids, the following basic relation is defined for them [29]:

(29) $$\sigma_{ij}=C_{ijkl}\varepsilon_{kl}$$

where $\sigma_{ij}$ and $\varepsilon_{kl}$ are the arrays of 2nd-order Cauchy stress and strain tensors, respectively; also, $C_{ijkl}$ represents the components of the 4th-order elasticity tensor of the material. Therefore, these relations can be modified as follows for each ply of the sandwich plates [27]:

(30) $${\left[\begin{array}{c} \sigma_{xx}\\ \sigma_{yy}\\ \sigma_{yz}\\ \sigma_{xz}\\ \sigma_{xy} \end{array}\right]}_{k}={\left[\begin{array}{ccccc} Q_{11} & Q_{12} & 0 & 0 & 0\\ Q_{12} & Q_{22} & 0 & 0 & 0\\ 0 & 0 & Q_{44} & 0 & 0\\ 0 & 0 & 0 & Q_{55} & 0\\ 0 & 0 & 0 & 0 & Q_{66} \end{array}\right]}_{k}\left[\begin{array}{c} \varepsilon_{xx}\\ \varepsilon_{yy}\\ \varepsilon_{yz}\\ \varepsilon_{xz}\\ \varepsilon_{xy} \end{array}\right],\;\;\;\;\;\;\;\;k=1,2,\ldots ,N$$

in which

(31) $$\begin{array}{l} Q_{11}=\frac{\hat{E}}{1-{\hat{\mu }}^{2}},\;\;\;\;Q_{12}=\hat{\mu }Q_{11},\;\;\;\;Q_{22}=Q_{11},\\ Q_{44}=Q_{55}=Q_{66}=\hat{G} \end{array}$$

Moreover, once the influence of GFs’ orientation angle is considered, the stress-strain relationship can be reformulated as below:

(32) $${\left[\begin{array}{c} \sigma_{xx}\\ \sigma_{yy}\\ \sigma_{yz}\\ \sigma_{xz}\\ \sigma_{xy} \end{array}\right]}_{k}={\left[\begin{array}{ccccc} {\bar{Q}}_{11} & {\bar{Q}}_{12} & 0 & 0 & {\bar{Q}}_{16}\\ {\bar{Q}}_{12} & {\bar{Q}}_{22} & 0 & 0 & {\bar{Q}}_{26}\\ 0 & 0 & {\bar{Q}}_{44} & 0 & 0\\ 0 & 0 & 0 & {\bar{Q}}_{55} & 0\\ {\bar{Q}}_{16} & {\bar{Q}}_{26} & 0 & 0 & {\bar{Q}}_{66} \end{array}\right]}_{k}\left[\begin{array}{c} \varepsilon_{xx}\\ \varepsilon_{yy}\\ \varepsilon_{yz}\\ \varepsilon_{xz}\\ \varepsilon_{xy} \end{array}\right],\;\;\;\;\;\;\;\;k=1,2,\ldots ,N$$

where

(33) $$\begin{array}{l} {\bar{Q}}_{11}=Q_{11}\cos^{4}\theta +2\left(Q_{12}+2Q_{66}\right)\sin^{2}\theta \cos^{2}\theta +Q_{22}\sin^{4}\theta ,\\ {\bar{Q}}_{12}=\left(Q_{11}+Q_{22}-4Q_{66}\right)\sin^{2}\theta \cos^{2}\theta +Q_{12}\left(\sin^{4}\theta +\cos^{4}\theta \right),\\ {\bar{Q}}_{16}=\left(Q_{11}-Q_{12}-2Q_{66}\right)\sin\theta \cos^{3}\theta +\left(Q_{12}-Q_{22}+2Q_{66}\right)\sin^{3}\theta \cos\theta ,\\ {\bar{Q}}_{22}=Q_{11}\sin^{4}\theta +2\left(Q_{12}+2Q_{66}\right)\sin^{2}\theta \cos^{2}\theta +Q_{22}\cos^{4}\theta ,\\ {\bar{Q}}_{26}=\left(Q_{11}-Q_{12}-2Q_{66}\right)\sin^{3}\theta \cos\theta +\left(Q_{12}-Q_{22}+2Q_{66}\right)\sin\theta \cos^{3}\theta ,\\ {\bar{Q}}_{66}=\left(Q_{11}+Q_{22}-2Q_{12}-2Q_{66}\right)\sin^{2}\theta \cos^{2}\theta +Q_{66}\left(\sin^{4}\theta +\cos^{4}\theta \right),\\ {\bar{Q}}_{44}={\bar{Q}}_{55}={\bar{Q}}_{66} \end{array}$$

In the above equations, θ is the orientation angle of GFs in the matrix of the composite, which is assumed to be determined with respect to *x*-axis; means, if θ=0, the GFs are distributed parallel to *x*-axis. Once the aforementioned definition is substituted in Equation (32), the stress resultants can be related to the material properties of the nanocomposite as below:

(34) $$\begin{array}{c} \left[\begin{array}{c} Nxx\\ Nyy\\ Nxy\\ M_{xx}^{b}\\ M_{yy}^{b}\\ M_{xy}^{b}\\ M_{xx}^{s}\\ M_{yy}^{s}\\ M_{xy}^{s} \end{array}\right]=\left[\begin{array}{ccccccccc} A_{11} & A_{12} & A_{16} & B_{11} & B_{12} & B_{16} & B_{11}^{s} & B_{12}^{s} & B_{16}^{s}\\ A_{12} & A_{22} & A_{26} & B_{12} & B_{22} & B_{26} & B_{12}^{s} & B_{22}^{s} & B_{26}^{s}\\ A_{16} & A_{26} & A_{66} & B_{16} & B_{26} & B_{66} & B_{16}^{s} & B_{26}^{s} & B_{66}^{s}\\ B_{11} & B_{12} & B_{16} & D_{11} & D_{12} & D_{16} & D_{11}^{s} & D_{12}^{s} & D_{16}^{s}\\ B_{12} & B_{22} & B_{26} & D_{12} & D_{22} & D_{26} & D_{12}^{s} & D_{22}^{s} & D_{26}^{s}\\ B_{16} & B_{26} & B_{66} & D_{16} & D_{26} & D_{66} & D_{16}^{s} & D_{26}^{s} & D_{66}^{s}\\ B_{11}^{s} & B_{12}^{s} & B_{16}^{s} & D_{11}^{s} & D_{12}^{s} & D_{16}^{s} & H_{11}^{s} & H_{12}^{s} & H_{16}^{s}\\ B_{12}^{s} & B_{22}^{s} & B_{26}^{s} & D_{12}^{s} & D_{22}^{s} & D_{26}^{s} & H_{12}^{s} & H_{22}^{s} & H_{26}^{s}\\ B_{16}^{s} & B_{26}^{s} & B_{66}^{s} & D_{16}^{s} & D_{26}^{s} & D_{66}^{s} & H_{16}^{s} & H_{26}^{s} & H_{66}^{s} \end{array}\right]\left[\begin{array}{c} \frac{\partial u}{\partial x}\\ \frac{\partial v}{\partial y}\\ \frac{\partial u}{\partial y}+\frac{\partial v}{\partial x}\\ -\frac{\partial^{2}w_{b}}{\partial x^{2}}\\ -\frac{\partial^{2}w_{b}}{\partial y^{2}}\\ -2\frac{\partial^{2}w_{b}}{\partial x\partial y}\\ -\frac{\partial^{2}w_{s}}{\partial \partial x^{2}}\\ -\frac{\partial^{2}w_{s}}{\partial y^{2}}\\ -2\frac{\partial^{2}w_{s}}{\partial x\partial y} \end{array}\right],\\ \left[\begin{array}{c} Q_{xz}\\ Q_{yz} \end{array}\right]=\left[\begin{array}{cc} A_{44}^{s} & 0\\ 0 & A_{55}^{s} \end{array}\right]\left[\begin{array}{c} \frac{\partial w_{s}}{\partial x}\\ \frac{\partial w_{s}}{\partial y} \end{array}\right] \end{array}$$

where through-the-thickness rigidities are:

(35) $$\begin{array}{l} \left[A_{n},B_{n},B_{n}^{s},D_{n},D_{n}^{s},H_{n}^{s}\right]=\sum_{k=1}^{N}\int_{h_{k}}^{h_{k+1}}\left[1,z,f\left(z\right),z^{2},zf\left(z\right),f^{2}\left(z\right)\right]Q_{n}\left(z\right)dz,\;\;\;\;\;\;n=\left(11,12,16,22,26,66\right)\\ \;\;\;\;\;\;\;\;\;\;\;\;\;\;\;\;\;\;\;\;\;\;\;\;\;\;\;\;\;\;\;\;\;\;A_{44}^{s}=A_{55}^{s}=\sum_{k=1}^{N}\int_{h_{k}}^{h_{k+1}}g^{2}\left(z\right)Gdz \end{array}$$

### 2.6. Governing Equations

Next, inserting Equation (34) in Equations (25)–(28), the governing equations of MSH nanocomposite plates can be enhanced. These equations can be seen by referring to Appendix C.

## 3. Analytical Solution

Many methods are known for the purpose of solving the static and dynamic problems in the field of structural mechanics [32,33,34,35,36,37]. This section is allocated to solve the final governing equations of the problem, presented in Equations (A1)–(A4), with the aid of an analytical method. To do so, a well-known wave method is implemented that has been discussed in detail in the book written by the two of the authors. Based on this approach, the wave solution of the displacement field of the system must be considered as follows [22]:

(36) $$\left\{\begin{array}{c} u\\ v\\ w_{b}\\ w_{s} \end{array}\right\}=\left\{\begin{array}{c} U\exp\left[i(\beta_{1}x+\beta_{2}y-\omega t)\right]\\ V\exp\left[i(\beta_{1}x+\beta_{2}y-\omega t)\right]\\ W_{b}\exp\left[i(\beta_{1}x+\beta_{2}y-\omega t)\right]\\ W_{s}\exp\left[i(\beta_{1}x+\beta_{2}y-\omega t)\right] \end{array}\right\}$$

where U, V, $W_{b}$, and $W_{s}$ are the unknown amplitudes of wave propagation. In addition, $\beta_{1}$ and $\beta_{2}$ are the wave numbers of wave propagation along *x* and *y* directions, respectively, and ω is the circular frequency of the dispersed waves. Once the above solution is inserted in Equations (A1)–(A4), the following eigenvalue equation will be obtained:

(37) $$K\Delta =M\omega^{2}\Delta$$

In above equation, K and M are stiffness and mass matrices, respectively. Additionally, Δ stands for the amplitude vector. Solving the above eigenvalue problem for ω gives the natural frequency of the propagated waves. For the sake of completeness, the arrays of stiffness and mass matrices are provided in Appendix B.

## 4. Numerical Results and Discussion

Herein, a series of numerical illustrations presented in order to investigate the effect of various parameters on the wave propagation behaviors of MSH nanocomposite plate. The nanocomposite plate is presumed to be made of epoxy, GF, and CNT in which material properties are chosen as the same as Refs. [20,38].

### 4.1. Validation

**Figure 2:**
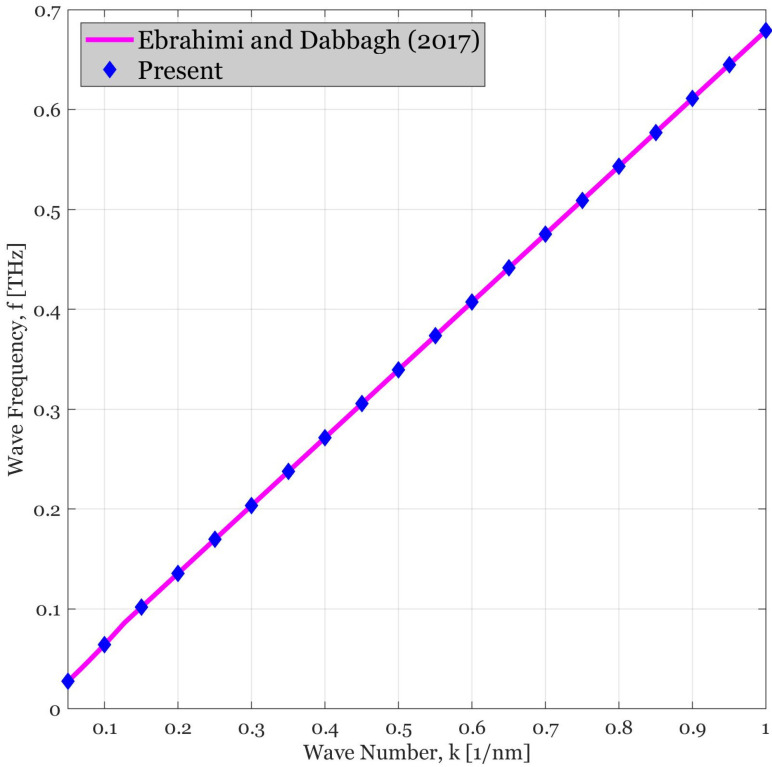
Comparison of the wave frequency versus wave number curve of the acoustic branch of waves dispersed in FG plates. The solid line is related to the response provided in [39] and the diamonds are the responses of present study.

The validity of the present methodology is examined in the framework of Figure 2. In this diagram, the dispersion curves provided by [39] are re-generated and the results are compared together. It can be observed that the dynamic responses are in an excellent agreement due to the fact that both studies undergo with the refined HSDT of the plates to probe the wave dispersion behaviors of the continuous systems. Therefore, the structural stiffness and inertia predicted by both methods are the same. It is worth mentioning that since both methods have revealed the wave response in small wave numbers, the obtained diagram seems to be a linear one which can be simulated with the aid of a linear regression in the form of y=mx+b.

### 4.2. Influence of Different Compositions on the Wave Propagation Response of the Continua

**Figure 3:**
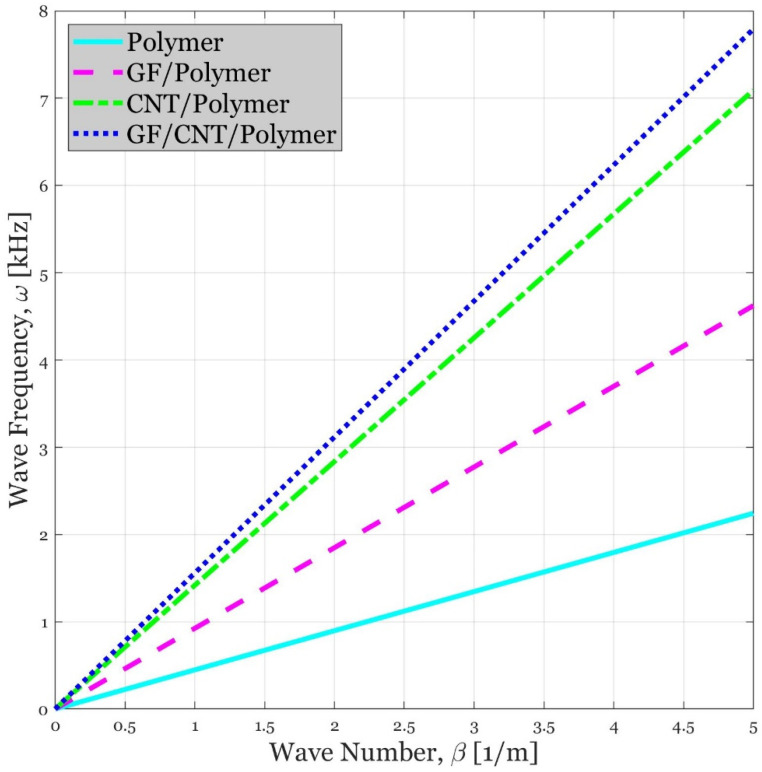
Comparison of acoustic wave frequency responses of sandwich plates fabricated from polymer, GF/polymer, CNT/polymer, and GF/CNT/polymer. α = 100 is employed in the cases which CNTs exist in the composition of the material. In this figure, the ${\left[0,30,45,60,90\right]}_{s}$ lay-up is used for the laminated composite.

Figure 3 is demonstrated in order to clarify the crucial role of material selection on the dynamic reactions of waves dispersed in a plate-type element. In this figure, plates composed of neat epoxy, GF-reinforced polymer (GFRP), CNTR polymer, and MSH nanocomposite are chosen to be compared together. It can be easily observed that the wave frequency of MSH nanocomposite possesses the peak value followed by CNTR polymer, GFRP, and pure polymer, respectively. The difference between wave frequency of these four types of plates can be better sensed in high wave numbers. When wave number exceeds β = 1 [1/m], for example, the wave frequency supported by MSH nanocomposite plates can be more than three times bigger than that of plates consisting of bare polymer. This trend can be attributed to the remarkable stiffness of nanocomposite material caused by the advanced material behaviors of tiny CNTs. Thanks to the aforementioned reason, MSH nanocomposites are better candidates for the purpose of achieving high acoustic responses among various types of composite structures that have been known to date.

### 4.3. Influence of Number of Plies on the Wave Propagation Response of the Continua

**Figure 4:**
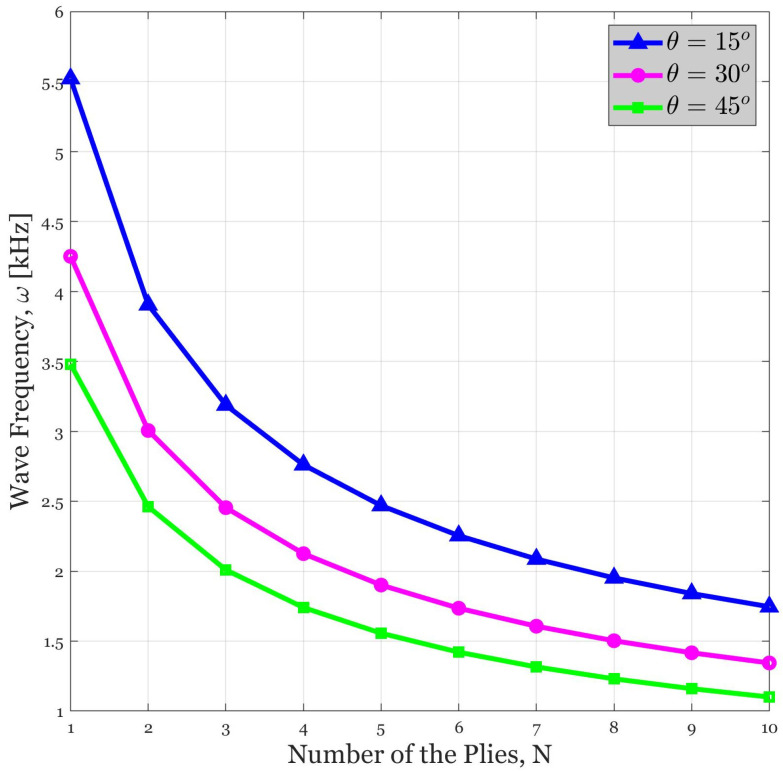
The variation in the wave frequency versus number of the plies for different amounts of the fibers’ orientation angle (β = 2).

Figure 4 is presented to exhibit the variation in the wave frequency against the number of the plies with respect to different values of the fibers’ orientation angle. According to this figure, an increase in the number of plies can be resulted in a decrease in the wave frequency. The major reason is that by increasing the number of plies the stiffness of the continuous system is decreased. Considering the direct relation between wave frequency and the stiffness, the following trend can be easily explained. In addition, in any specific number of piles, the greatest wave frequency corresponds with θ=15. Thus, it can be concluded that the smaller the angle with respect to *x*-axis is, the greater the wave frequency will be. As another interesting feature of this diagram, it must be mentioned that the effect of orientation angle of the fibers is not dependent on the number of the plies.

### 4.4. Influence of Volume Fraction of the GFs on the Wave Propagation Response of the Continua for Different Lay-Ups

**Figure 5:**
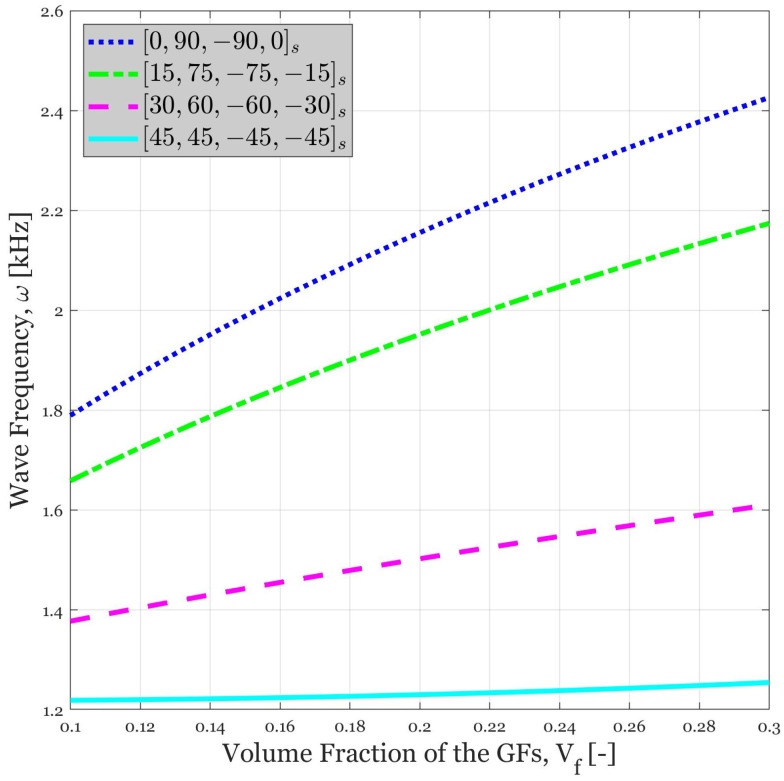
The variation in the wave frequency versus volume fraction of the GFs for various lay-ups of eight-layered laminates (β = 2).

In another illustration (see Figure 5), the impact of volume fraction of the GFs for different lay-ups on the frequency of the dispersed waves is investigated. It can be well observed that utilizing the higher GFs’ volume fraction results in amplification of the dynamic response. From a physical point of view, this trend can be verified by considering the fact that the stiffness can be improved by an increase in the volume fraction of the reinforcing fibers, which results in an increase in the wave frequency. In addition to this fact, the importance of selecting suitable lay-up is illustrated, too. It is noteworthy that there is a remarkable difference between various lay-ups. As shown in Figure 5, the highest value of frequency belongs to [0, 90, –90, 0]_s_ lay-up which corresponds to the case that GFs are parallel to the *x*-axis and *y*-axis, respectively. The reason for this can be explained throughout Equation (33) that each parameter has its maximum value while [0, 90, –90, 0]_s_ lay-up is used. The more the deviation from this lay-up, the more the wave frequency will be reduced. The ultimate effect of this kind of stiffness reduction can be observed in [45, 45, –45, –45]_s_ lay-up where wave frequency has plateaued on a little above 1.2 kHz for all of the considered volume fractions of GFs.

### 4.5. Influence of Weight Fraction of the CNTs on the Wave Propagation Response of the Continua for Various Length-to-Diameter Ratios of CNTs

**Figure 6:**
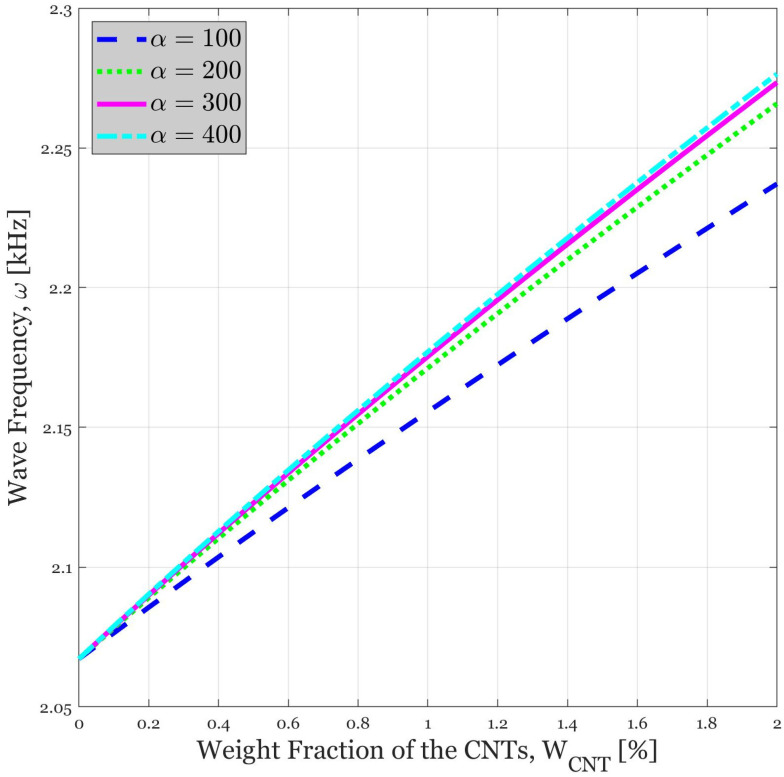
Variation in the wave frequency versus weight fraction of the CNTs for various CNTs’ length-to-diameter ratio. In this diagram, [0, 90, −90, 0]_s_ lay-up is used (V_f_ = 0.2, β = 2).

Figure 6 is plotted to show the effect of different length-to-diameter ratios of the CNTs on the wave frequency while the weight fraction is assumed to be changed. It can be perceived that an increase in the length-to-diameter ratio leads to higher wave frequencies. The justification for this pattern is the suitability of extremely slender CNTs to be used as reinforcements in composite materials [40,41]. The nanocomposite can then be improved more effectively if CNTs with large length-to-diameter ratios are used in the manufacturing process. Moreover, the effect of the weight fractions of CNTs is highlighted. As it was mentioned in the previous section, adding reinforcement components enhances the stiffness, and because of the direct relation between stiffness and frequency, it has an outstanding effect on the wave frequency. It is mentionable that the impact of the weight fraction of CNTs is greater than the length-to-diameter ratio. A two percent raise in the weight fraction of the CNTs results in a 0.2 kHz enhancement in the wave frequency, whereas, using α = 400 instead of α = 100 can, in the best situation, generate an improvement of 0.03 kHz.

## 5. Concluding Remarks

This paper was arranged to compensate for the lack of reliable data about the wave propagation problem of three-phase GF/CNT/polymer hybrid nanocomposites plates. Herein, the most magnificent highlights of this study are summarized.

- Since an increase in the number of plies has a downside effect on the wave frequency, it is best to find the optimal number to satisfy the design criteria.
- The MSH nanocomposite plate can achieve its greatest dynamic response once the ${\left[0,90,-90,0\right]}_{s}$ lay-up is employed.
- Adding either the weight fraction of the CNTs or the volume fraction of the GFs amplifies the natural frequency of the propagated waves.

Regarding these facts, MSH nanocomposite structures with a large reinforcing load can be well-suited to the high-frequency devices demanded by engineering applications. Another way to fulfill the desired outcome is to increase the length-to-diameter ratio of the reinforcing CNTs. However, if the latter is pursued, the designer needs to care about potential effect of the wavy shape of nanotubes which might reduce the stiffness of the nanocomposite significantly.

## Appendix A

The arrays of Eshelby tensor used in Section 2.2 are:

$$\begin{array}{l} S_{1111}=\frac{1}{2\left(1-\mu_{m}\right)}\left(1-2\mu_{m}+\frac{3\alpha^{2}-1}{\alpha^{2}-1}-\left[1-2\mu_{m}+\frac{3\alpha^{2}}{\alpha^{2}-1}\right]\tilde{J}\right),\\ S_{2222}=\frac{3}{8\left(1-\mu_{m}\right)}\frac{\alpha^{2}}{\alpha^{2}-1}+\frac{1}{4\left(1-\mu_{m}\right)}\left(1-2\mu_{m}-\frac{9}{4\left(\alpha^{2}-1\right)}\right)\tilde{J},\\ S_{2233}=\frac{1}{4\left(1-\mu_{m}\right)}\left(\frac{\alpha^{2}}{2\left(\alpha^{2}-1\right)}-\left[1-2\mu_{m}-\frac{3}{4\left(\alpha^{2}-1\right)}\right]\tilde{J}\right),\\ S_{2211}=-\frac{1}{2\left(1-\mu_{m}\right)}\frac{\alpha^{2}}{\alpha^{2}-1}+\frac{1}{4\left(1-\mu_{m}\right)}\left(\frac{3\alpha^{2}}{\alpha^{2}-1}-\left[1-2\mu_{m}\right]\right)\tilde{J},\\ S_{1122}=-\frac{1}{2\left(1-\mu_{m}\right)}\left(1-2\mu_{m}+\frac{1}{\alpha^{2}-1}\right)+\frac{1}{2\left(1-\mu_{m}\right)}\left(1-2\mu_{m}+\frac{3}{2\left(\alpha^{2}-1\right)}\right)\tilde{J},\\ S_{2323}=\frac{1}{4\left(1-\mu_{m}\right)}\left(\frac{\alpha^{2}}{2\left(\alpha^{2}-1\right)}+\left[1-2\mu_{m}-\frac{3}{4\left(\alpha^{2}-1\right)}\right]\tilde{J}\right),\\ S_{1212}=\frac{1}{4\left(1-\mu_{m}\right)}\left(1-2\mu_{m}-\frac{\alpha^{2}+1}{\alpha^{2}-1}-0.5\left[1-2\mu_{m}-\frac{3\left(\alpha^{2}+1\right)}{\alpha^{2}-1}\right]\tilde{J}\right) \end{array}$$

where

$$\tilde{J}=\frac{\alpha^{2}\sqrt{\alpha^{2}-1}-\alpha \cosh^{-1}\alpha }{\left(\alpha^{2}-1\right)\sqrt{\alpha^{2}-1}}$$

## Appendix B

The arrays of stiffness and mass symmetric matrices, used in Equation (37), are as below:

$$\begin{array}{l} k_{11}=-A_{11}\beta_{1}^{2}-2A_{16}\beta_{1}\beta_{2}-A_{66}\beta_{2}^{2},\\ k_{12}=-A_{16}\beta_{1}^{2}-\left(A_{12}+A_{66}\right)\beta_{1}\beta_{2}-A_{26}\beta_{2}^{2},\\ k_{13}=iB_{11}\beta_{1}^{3}+3iB_{16}\beta_{1}^{2}\beta_{2}+i\left(B_{12}+2B_{66}\right)\beta_{1}\beta_{2}^{2}+iB_{26}\beta_{2}^{3},\\ k_{14}=iB_{11}^{s}\beta_{1}^{3}+3iB_{16}^{s}\beta_{1}^{2}\beta_{2}+i\left(B_{12}^{s}+2B_{66}^{s}\right)\beta_{1}\beta_{2}^{2}+iB_{26}^{s}\beta_{2}^{3},\\ k_{21}=-A_{16}\beta_{1}^{2}-\left(A_{12}+A_{66}\right)\beta_{1}\beta_{2}-A_{26}\beta_{2}^{2},\\ k_{22}=-A_{66}\beta_{1}^{2}-2A_{26}\beta_{1}\beta_{2}-A_{22}\beta_{2}^{2},\\ k_{23}=iB_{16}\beta_{1}^{3}+i\left(B_{12}+2B_{66}\right)\beta_{1}^{2}\beta_{2}+3iB_{26}\beta_{1}\beta_{2}^{2}+iB_{22}\beta_{2}^{3},\\ k_{24}=iB_{16}^{s}\beta_{1}^{3}+i\left(B_{12}^{s}+2B_{66}^{s}\right)\beta_{1}^{2}\beta_{2}+3iB_{26}^{s}\beta_{1}\beta_{2}^{2}+iB_{22}^{s}\beta_{2}^{3},\\ k_{31}=-iB_{11}\beta_{1}^{3}-3iB_{16}\beta_{1}^{2}\beta_{2}-i\left(B_{16}+2B_{66}\right)\beta_{1}\beta_{2}^{2}-iB_{26}\beta_{2}^{3},\\ k_{32}=-iB_{16}\beta_{1}^{3}-i\left(B_{12}+2B_{66}\right)\beta_{1}^{2}\beta_{2}-3iB_{26}\beta_{1}\beta_{2}^{2}-iB_{22}\beta_{2}^{3},\\ k_{33}=-D_{11}\beta_{1}^{4}-4D_{16}\beta_{1}^{3}\beta_{2}-2\left(D_{12}+2D_{66}\right)\beta_{1}^{2}\beta_{2}^{2}-4D_{26}\beta_{1}\beta_{2}^{3}-D_{22}\beta_{2}^{4},\\ k_{34}=-D_{11}^{s}\beta_{1}^{4}-4D_{16}^{s}\beta_{1}^{3}\beta_{2}-2\left(D_{12}^{s}+2D_{66}^{s}\right)\beta_{1}^{2}\beta_{2}^{2}-4D_{26}^{s}\beta_{1}\beta_{2}^{3}-D_{26}^{s}\beta_{2}^{4},\\ k_{41}=-iB_{11}^{s}\beta_{1}^{3}-3iB_{16}^{s}\beta_{1}^{2}\beta_{2}-i\left(B_{12}^{s}+2B_{66}^{s}\right)\beta_{1}\beta_{2}^{2}-iB_{26}^{s}\beta_{2}^{3},\\ k_{42}=-iB_{16}^{s}\beta_{1}^{3}-i\left(B_{12}^{s}+2B_{66}^{s}\right)\beta_{1}^{2}\beta_{2}-3iB_{26}^{s}\beta_{1}\beta_{2}^{2}-iB_{22}^{s}\beta_{2}^{3},\\ k_{43}=-D_{11}^{s}\beta_{1}^{4}-4D_{16}^{s}\beta_{1}^{3}\beta_{2}-2\left(D_{12}^{s}+D_{66}^{s}\right)\beta_{1}^{2}\beta_{2}^{2}-4D_{26}^{s}\beta_{1}\beta_{2}^{3}-D_{22}^{s}\beta_{2}^{4},\\ k_{44}=-H_{11}^{s}\beta_{1}^{4}-4H_{16}^{s}\beta_{1}^{3}\beta_{2}-2\left(H_{12}^{s}+H_{66}^{s}\right)\beta_{1}^{2}\beta_{2}^{2}-4H_{26}^{s}\beta_{1}\beta_{2}^{3}-H_{22}^{s}\beta_{2}^{4}-\left(\beta_{1}^{2}+\beta_{2}^{2}\right)A_{44}^{s} \end{array}$$

and

$$\begin{array}{l} m_{11}=-I_{0},\;\;m_{12}=0,\;\;m_{13}=-iI_{1}\beta_{1},\;\;m_{14}=-iJ_{1}\beta_{1},\\ m_{21}=0,\;\;m_{22}=-I_{0},\;\;m_{23}=-iI_{0}\beta_{2},\;\;m_{24}=-iJ_{1}\beta_{2},\\ m_{31}=-iI_{1}\beta_{1},\;\;m_{32}=-iI_{1}\beta_{2},\;\;m_{33}=\left(I_{0}+I_{2}\left(\beta_{1}^{2}+\beta_{2}^{2}\right)\right),\;\;m_{34}=\left(I_{0}+J_{2}\left(\beta_{1}^{2}+\beta_{2}^{2}\right)\right),\\ m_{41}=-iJ_{1}\beta_{1},\;\;m_{42}=-iJ_{1}\beta_{2},\;\;m_{43}=\left(I_{0}+J_{2}\left(\beta_{1}^{2}+\beta_{2}^{2}\right)\right),\;\;m_{44}=\left(I_{0}+K_{2}\left(\beta_{1}^{2}+\beta_{2}^{2}\right)\right), \end{array}$$

## Appendix C

The governing equations of the problem (introduced in Section 2.6) can be expressed as follows:

(A1) $$\begin{array}{l} A_{11}\frac{\partial^{2}u}{\partial x^{2}}+2A_{16}\frac{\partial^{2}u}{\partial x\partial y}+A_{66}\frac{\partial^{2}u}{\partial y^{2}}+A_{16}\frac{\partial^{2}v}{\partial x^{2}}+\left(A_{12}+A_{66}\right)\frac{\partial^{2}v}{\partial x\partial y}+A_{26}\frac{\partial^{2}y}{\partial y^{2}}\\ -B_{11}\frac{\partial^{3}w_{b}}{\partial x^{3}}-3B_{16}\frac{\partial^{3}w_{b}}{\partial x^{2}\partial y}-\left(B_{12}+2B_{66}\right)\frac{\partial^{3}w_{b}}{\partial x\partial y^{2}}-B_{26}\frac{\partial^{3}w_{b}}{\partial y^{3}}-B_{11}^{s}\frac{\partial^{3}w_{s}}{\partial x^{3}}-\\ 3B_{16}^{s}\frac{\partial^{3}w_{s}}{\partial x^{2}\partial y}-\left(B_{12}^{s}+B_{66}^{s}\right)\frac{\partial^{3}w_{s}}{\partial x\partial y^{2}}-B_{26}^{s}\frac{\partial^{3}w_{s}}{\partial y^{3}}-I_{0}\frac{\partial^{2}u}{\partial t^{2}}+I_{1}\frac{\partial^{3}w_{b}}{\partial x\partial t^{2}}+J_{1}\frac{\partial^{3}w_{b}}{\partial x\partial t^{2}}=0 \end{array}$$

(A2) $$\begin{array}{l} A_{16}\frac{\partial^{2}u}{\partial x^{2}}+\left(A_{12}+A_{66}\right)\frac{\partial^{2}u}{\partial x\partial y}+A_{26}\frac{\partial^{2}u}{\partial y^{2}}+A_{66}\frac{\partial^{2}v}{\partial x^{2}}+2A_{26}\frac{\partial^{2}v}{\partial x\partial y}+A_{22}\frac{\partial^{2}v}{\partial y^{2}}\\ -B_{16}\frac{\partial^{3}w_{b}}{\partial x^{3}}-\left(B_{12}+2B_{66}\right)\frac{\partial^{3}w_{b}}{\partial x^{2}\partial y}-3B_{26}\frac{\partial^{3}w_{b}}{\partial x\partial y^{2}}-B_{22}\frac{\partial^{3}w_{b}}{\partial y^{3}}-B_{16}^{s}\frac{\partial^{3}w_{s}}{\partial x^{3}}-\\ \left(B_{12}^{s}+2B_{66}^{s}\right)\frac{\partial^{3}w_{s}}{\partial x^{2}\partial y}-3B_{26}^{s}\frac{\partial^{3}w_{b}}{\partial x\partial y^{2}}-B_{22}^{s}\frac{\partial^{3}w_{b}}{\partial y^{3}}-I_{0}\frac{\partial^{2}v}{\partial t^{2}}+I_{1}\frac{\partial^{3}w_{b}}{\partial y\partial t^{2}}+J_{1}\frac{\partial^{3}w_{b}}{\partial y\partial t^{2}}=0 \end{array}$$

(A3) $$\begin{array}{l} B_{11}\frac{\partial^{3}u}{\partial x^{3}}+3B_{16}\frac{\partial^{3}u}{\partial x^{2}\partial y}+\left(B_{12}+2B_{66}\right)\frac{\partial^{3}u}{\partial x\partial y^{2}}+B_{26}\frac{\partial^{3}u}{\partial y^{3}}+B_{16}\frac{\partial^{3}v}{\partial x^{3}}+\left(B_{12}+2B_{66}\right)\frac{\partial^{3}v}{\partial x^{2}\partial y}+\\ 3B_{26}\frac{\partial^{3}v}{\partial x\partial y^{2}}+B_{22}\frac{\partial^{3}v}{\partial y^{3}}-D_{11}\frac{\partial^{4}w_{b}}{\partial x^{4}}-4D_{16}\frac{\partial^{4}w_{b}}{\partial x^{3}\partial y}-2\left(D_{12}+2D_{66}\right)\frac{\partial^{4}w_{b}}{\partial x^{2}\partial y^{2}}-4D_{26}\frac{\partial^{4}w_{b}}{\partial x\partial y^{3}}\\ -D_{22}\frac{\partial^{4}w_{b}}{\partial y^{4}}-D_{11}^{s}\frac{\partial^{4}w_{s}}{\partial x^{4}}-4D_{16}^{s}\frac{\partial^{4}w_{s}}{\partial x^{3}\partial y}-2\left(D_{12}^{s}+2D_{66}^{s}\right)\frac{\partial^{4}w_{s}}{\partial x^{2}\partial y^{2}}-4D_{26}^{s}\frac{\partial^{4}w_{b}}{\partial x\partial y^{3}}-D_{22}^{s}\frac{\partial^{4}w_{s}}{\partial y^{4}}\\ -I_{0}\frac{\partial^{2}\left(w_{b}+w_{s}\right)}{\partial t^{2}}-I_{1}\left(\frac{\partial^{3}u}{\partial x\partial t^{2}}+\frac{\partial^{3}v}{\partial y\partial t^{2}}\right)+I_{2}\left(\frac{\partial^{4}w_{b}}{\partial x^{2}\partial t^{2}}+\frac{\partial^{4}w_{b}}{\partial x^{2}\partial t^{2}}\right)+J_{2}\left(\frac{\partial^{4}w_{s}}{\partial x^{2}\partial t^{2}}+\frac{\partial^{4}w_{s}}{\partial y^{2}\partial t^{2}}\right)=0 \end{array}$$

(A4) $$\begin{array}{l} B_{11}^{s}\frac{\partial^{3}u}{\partial x^{3}}+3B_{16}^{s}\frac{\partial^{3}u}{\partial x^{2}\partial y}+\left(B_{12}^{s}+2B_{66}^{s}\right)\frac{\partial^{3}u}{\partial x\partial y^{2}}+B_{26}^{s}\frac{\partial^{3}u}{\partial y^{3}}+B_{16}^{s}\frac{\partial^{3}v}{\partial x^{3}}+\left(B_{12}^{s}+2B_{66}^{s}\right)\frac{\partial^{3}v}{\partial x^{2}\partial y}\\ +3B_{26}^{s}\frac{\partial^{3}v}{\partial x\partial y^{2}}+B_{22}^{s}\frac{\partial^{3}v}{\partial y^{3}}-D_{11}^{s}\frac{\partial^{4}w_{b}}{\partial x^{4}}-4D_{16}^{s}\frac{\partial^{4}w_{b}}{\partial x^{3}\partial y}-2\left(D_{12}^{s}+2D_{66}^{s}\right)\frac{\partial^{4}w_{b}}{\partial x^{2}\partial y^{2}}-4D_{26}^{s}\frac{\partial^{4}w_{b}}{\partial x\partial y^{3}}\\ -D_{22}^{s}\frac{\partial^{4}w_{b}}{\partial y^{4}}-H_{11}^{s}\frac{\partial^{4}w_{s}}{\partial x^{4}}-4H_{16}^{s}\frac{\partial^{4}w_{s}}{\partial x^{3}\partial y}-2\left(H_{12}^{s}+2H_{66}^{s}\right)\frac{\partial^{4}w_{s}}{\partial x^{2}\partial y^{2}}-4H_{26}^{s}\frac{\partial^{4}w_{b}}{\partial x\partial y^{3}}-H_{22}^{s}\frac{\partial^{4}w_{s}}{\partial y^{4}}\\ +A_{44}^{s}\left(\frac{\partial^{2}w_{s}}{\partial x^{2}}+\frac{\partial^{2}w_{s}}{\partial y^{2}}\right)-I_{0}\frac{\partial^{2}\left(w_{b}+w_{s}\right)}{\partial t^{2}}-J_{1}\left(\frac{\partial^{3}u}{\partial x\partial t^{2}}+\frac{\partial^{3}v}{\partial y\partial t^{2}}\right)+J_{2}\left(\frac{\partial^{4}w_{b}}{\partial x^{2}\partial t^{2}}+\frac{\partial^{4}w_{b}}{\partial x^{2}\partial t^{2}}\right)+\\ K_{2}\left(\frac{\partial^{4}w_{s}}{\partial x^{2}\partial t^{2}}+\frac{\partial^{4}w_{s}}{\partial y^{2}\partial t^{2}}\right)=0 \end{array}$$
